# Optimization of the Chemical Monoubiquitination System for Low-Solubility Protein: Achieving Balance Between Specificity and Yield

**DOI:** 10.3390/cimb48070666

**Published:** 2026-06-29

**Authors:** Qingyu Cao, Mengyuan Zhang, Dan Wang, Kaixuan He, Yuanyuan Mei, Ning Ning Wang

**Affiliations:** 1 Tianjin Key Laboratory of Protein Sciences, Department of Plant Biology and Ecology, College of Life Sciences, Nankai University, Tianjin 300071, China; 2 State Key Laboratory of North China Crop Improvement and Regulation/Key Laboratory of Hebei Province for Plant Physiology and Molecular Pathology, College of Life Sciences, Hebei Agricultural University, Baoding 071001, China

**Keywords:** monoubiquitination, chemical synthesis, SSPP, low-solubility protein

## Abstract

Monoubiquitination is a significant post-translational modification that plays a pivotal role in various biological processes. Chemical monoubiquitination holds significant value in investigating the functional implications of site-specific ubiquitination on target proteins. Despite all progress made in this area, conventional enzymatic methods so far rely largely on high yields of substrate proteins and the removal of tags to prevent non-specific ubiquitin binding, which poses substantial challenges for low-solubility proteins. Here, an optimized chemical monoubiquitination system that facilitates precise, site-specific ubiquitination of low-solubility target protein was developed using SSPP as an example. A cysteine-free GST tag (GST^4CS^) was engineered, and a flexible (GGGGS)_3_ linker was incorporated to mitigate steric hindrance and enhance the solubility of GST-SSPP fusion protein, resulting in a 2.5-fold increase in purification yield. Successful monoubiquitination of SSPP at the position of lysine 305 was achieved using disulfide-mediated conjugation, as proven via SDS-PAGE and Western blotting. Moreover, the phosphatase assay showed that monoubiquitination at residue C305 of the mutated SSPP significantly decreased its phosphatase activity. This system eliminates tag interference and enhances compatibility with low-solubility targets, providing a robust platform for functional studies of plant protein ubiquitination.

## 1. Introduction

Protein ubiquitination involves the attachment of ubiquitin to other proteins, thereby serving as a critical form of post-translational modification [1,2,3,4]. Among the various forms of ubiquitination, monoubiquitination stands out as a multifaceted regulator of protein function and localization, highlighting its functional complexity in cellular processes [5,6,7]. For example, the monoubiquitination of the human guanine nucleotide-binding protein KRAS at Lysine104 and Lysine147 modulates its dynamics and interaction with partner proteins during cell growth regulation [8]. In addition, monoubiquitination can alter protein localization and stability. The human E3 ubiquitin ligase neural precursor cell expressed, developmentally down-regulated 4-1 (NEDD4-1) catalyzes the monoubiquitination of phosphatase and tensin homolog deleted on chromosome 10 (PTEN), a protein tyrosine phosphatase, through its (homologous to the E6-AP Carboxyl Terminus) HECT domain, primarily targeting two lysine residues (Lys13 and Lys289). This modification promotes the nuclear import of PTEN, thus protecting it from degradation [9,10]. Recent studies have also demonstrated that the cytoplasmic receptor kinase BOTRYTIS-INDUCED KINASE 1 (BIK1), upon microbial detection, is phosphorylated by BRI1-ASSOCIATED KINASE 1 (BAK1) and subsequently monoubiquitinated by *Arabidopsis Tóxicos en Levadura* 44/45 (ATL44/45). This monoubiquitination enables the dissociation of BIK1 from the FLS1-BAK2-BIK1 complex, facilitating its endocytosis [11]. Recently, the localization and activity of abscisic acid (ABA) receptors in *Arabidopsis* were found to be regulated by a novel E3 ligase DEGRADATION OF ALPHA2 10A (DOA10A) through monoubiquitination, leading to enhanced ABA perception [12].

The chemical monoubiquitination represents a crucial technique for exploring the functional consequences of site-specific ubiquitination on target proteins [13,14,15]. A widely used chemical approach for monoubiquitination replaces the isopeptide bond with a disulfide bond by substituting a target lysine with cysteine in the protein, allowing site-specific linkage to ubiquitin^G76C^. This technique was pioneered by Merkley et al. (2005) for stabilizing the E2–ubiquitin complex [16]. Subsequently, Baker’s team achieved an 80% monoubiquitination rate but faced challenges in protein separation [17,18]. Shin et al. (2017) further enhanced the efficiency to 95% using iterative ubiquitin^G76C^ additions and CuCl_2_-catalyzed oxidation and developed an effective size-exclusion chromatography method for separating monoubiquitinated Rab5 from unmodified proteins [19].

Despite the progress made in the chemical monoubiquitination, all methods so far, including the one established by Shin et al. (2017), heavily rely on the high yields of substrate proteins [19]. In the meantime, the purification tag of the target protein is often removed in chemical ubiquitination protocols to eliminate the non-specific binding of ubiquitin to any potential cysteine residue within the tag itself [20,21,22]. However, this approach poses significant challenges for low-solubility proteins. On one hand, the yield of purified protein may be very low; on the other, the large tags used for the purification of poorly soluble proteins cannot be removed, as they are crucial for enhancing the protein’s solubility. This presents a dilemma in the purification and subsequent ubiquitination of such proteins.

Senescence-suppressed protein phosphatase (SSPP, At5g02760), a member of the PP2C protein phosphatase family form *Arabidopsis thaliana*, was selected for this study because it represents a biologically relevant target of monoubiquitination while also posing substantial technical challenges for biochemical analysis. Previous studies have shown that SSPP undergoes monoubiquitination and exhibits relatively poor solubility during recombinant expression and purification [23,24,25]. In addition, as an active protein phosphatase, its enzymatic activity can be readily evaluated following ubiquitin conjugation. Together, these characteristics make SSPP a stringent test substrate for assessing strategies aimed at improving site-specific protein monoubiquitination under conditions where protein solubility and recovery are limiting factors. Using SSPP as a representative low-solubility substrate, we optimized the chemical monoubiquitination workflow to enhance conjugation efficiency, minimize interference from tag-derived cysteine residues, and improve product yield while maintaining modification specificity. This optimized approach provides an applicable strategy for the monoubiquitination modification of challenging protein substrates.

## 2. Methods and Materials

### 2.1. Preparation of Ubiquitin–Cysteamine (Ub-SH)

#### 2.1.1. Construction of Ubiquitin^G76C^ Expression Plasmid

To construct His-Ubiquitin^G76C^, the Ubiquitin^G76C^ fragment was amplified using 28a-UBG76C-F/R as the forward and reverse primers, respectively, with the ubiquitin sequence synthesized by BGI Genomics as the template. The primer sequences and template sequence are provided in Supplementary Tables S1 and S2, respectively. PCR amplification was performed using KOD One PCR Master Mix (TOYOBO Co., Ltd., Osaka, Japan). In parallel, the pET28a vector was digested with BamH I and Not I to generate the linearized vector backbone. The PCR product and the linearized vector fragment were purified using an Axygen^®^ AxyPrep DNA Gel Extraction Kit (AP-GX-50, Axygen Biosciences, Union City, CA, USA), and then assembled using a Beyotime Seamless Cloning Kit (D7010S, Beyotime Biotechnology, Shanghai, China) according to the manufacturer’s instructions. The resulting plasmid was confirmed by Sanger sequencing (BGI Genomics, Beijing, China). The nucleotide sequences of all expressed proteins and fusion constructs used in this study are provided in Supplementary Table S3.

Unless otherwise specified, all DNA fragments used in this study were amplified using the following PCR system. Each 50 μL PCR reaction contained 25 μL of KOD One PCR Master Mix, 0.3 μM of each primer, template DNA, and nuclease-free water. The ultrapure water used throughout the experiments was prepared using a Milli-Q water purification system and had a resistivity of ≥18.2 MΩ·cm at 25 °C. PCR amplification was performed under the following conditions: initial denaturation at 98 °C for 10 s; 30 cycles of denaturation at 98 °C for 10 s, annealing at 55–65 °C for 5 s depending on the primer melting temperature, and extension at 68 °C for 5–10 s/kb; followed by a final extension at 68 °C for 1 min.

#### 2.1.2. Transformation into *Escherichia coli* (*E. coli*) Transetta (DE3) Cells

For protein expression, the plasmid was transformed into *E. coli* Transetta (DE3) competent cells.

#### 2.1.3. Strain Activation

A single colony was inoculated into 5 mL LB medium (10 g/L tryptone, 5 g/L yeast extract, and 10 g/L NaCl) supplemented with 100 mg/L kanamycin (Kan) and 17 mg/L chloramphenicol (Chl), and cultured overnight at 37 °C with shaking in a 2 L Erlenmeyer flask at 210 rpm.

#### 2.1.4. Large-Scale Culture

The culture was diluted 1:100 into 400 mL fresh LB medium (100 mg/L Kan + 17 mg/L Chl) and incubated at 37 °C with shaking in a 2 L Erlenmeyer flask at 210 rpm using a shaking amplitude (orbit diameter) of 25 mm.

#### 2.1.5. Protein Induction

When OD_600_ of bacterial suspension reached 0.6–0.8 (~3.5 h), 1 mL of culture was collected, centrifuged at 13,000× *g* for 1 min, and resuspended in lysis buffer (1× phosphate-buffered saline (PBS) + 20 mM imidazole). The 1× PBS used in this study consisted of 137 mM NaCl, 2.7 mM KCl, 10 mM Na_2_HPO_4_, and 2 mM KH_2_PO_4_ (pH 7.4). The sample was mixed with 5× loading buffer (250 mM Tris-HCl (pH 6.8), 100 g/L sodium dodecyl sulfate (SDS), 5 g/L bromophenol blue (BPB), 50% (*v*/*v*) glycerol, and 5% (*v*/*v*) β-mercaptoethanol); boiled at 100 °C for 10 min; and immediately subjected to SDS–PAGE analysis as a pre-induction control. Isopropyl β-D-1-thiogalactopyranoside (IPTG) was added to the remaining culture at a final concentration of 0.6 mM, followed by induction at 16 °C for 20 h.

#### 2.1.6. Cell Harvest

Cells harvested from 400 mL bacterial culture were pelleted via centrifugation at 7000× *g* (4 °C, 10 min), resuspended in 20 mL pre-chilled binding buffer (1× PBS + 20 mM imidazole), and supplemented with 1 mM phenylmethylsulfonyl fluorid (PMSF) from a 100 mM stock solution prepared in isopropanol and stored at −20 °C. Aliquots (20 mL/tube) were flash-frozen in liquid nitrogen and stored at −80 °C.

#### 2.1.7. Cell Lysis

Frozen cells were thawed in a 37 °C water bath and immediately placed on ice. Cell lysates were disrupted using a JY92-II ultrasonic homogenizer (Ningbo Scientz Biotechnology Co., Ltd., Ningbo, Zhejiang, China) at 300 W on ice, using a pulse program of 2 s sonication followed by 5 s intervals for a total of 15 cycles. A post-sonication sample was collected, centrifuged, resuspended in loading buffer, boiled, and analyzed.

#### 2.1.8. Centrifugation and Filtration

The lysate was centrifuged at 13,000× *g* (4 °C, 30 min) to remove insoluble debris, and the supernatant was filtered through a 0.45 μm polyethersulfone (PES) syringe filter.

#### 2.1.9. Affinity Chromatography (His Trap FF Column)

The filtered supernatant was loaded onto a 1 mL His Trap™ FF column (Cytiva, Marlborough, MA, USA) pre-equilibrated with 10 column volumes (CVs) of binding buffer at 4 °C. The flow-through was collected for analysis (flow rate: 0.2–1 mL/min).

#### 2.1.10. Column Washing

The column was washed with >20 CVs of binding buffer until the A280 (Nanodrop 2000, Thermo Fisher Scientific, Waltham, MA, USA) matched the baseline or no residual protein was detected in 5 μL wash fractions using a Bradford protein assay reagent (PA102, TIANGEN Biotech, Beijing, China).

#### 2.1.11. Protein Elution

After washing, the column was incubated briefly with buffer containing 200 mM imidazole prior to elution on ice. The target protein was subsequently eluted, and a total eluate volume of 3 mL was collected for the next step. Protein concentrations were determined using the Bradford assay. The total protein yield was calculated based on the measured protein concentration and final elution volume, and the purification yield was expressed as milligrams of purified protein per liter of bacterial culture. The purification yields of the relevant protein constructs are shown in Supplementary Table S4.

#### 2.1.12. Ultrafiltration (Amicon^®^ Ultra 3K)

The eluted protein was concentrated using a 3 kDa molecular weight cutoff (MWCO) ultrafiltration device equipped with regenerated cellulose membranes (Merck Millipore, Burlington, MA, USA), which had been pre-treated with reducing buffer (2 mM tris(2-carboxyethyl) phosphine (TCEP), 50 mM Tris-HCl, pH 7.5, and 150 mM NaCl).

#### 2.1.13. Buffer Exchange

The protein was buffer-exchanged into reducing buffer through three cycles of dilution and concentration using a 3 kDa MWCO ultrafiltration device. In each cycle, 10 mL of reducing buffer was added for buffer exchange. After three rounds of buffer replacement, the protein solution was concentrated to a final volume of approximately 500 μL, depending on the concentration of the target protein, to ensure that the final protein concentration was at least 10 mg/mL.

#### 2.1.14. SDS-PAGE and Coomassie Brilliant Blue Staining

Proteins were separated on 12% SDS–polyacrylamide gels using the Tris–glycine buffer system (Laemmli system) by a Mini-PROTEAN Tetra Vertical Electrophoresis Cell system (Bio-Rad Laboratories, Hercules, CA, USA). The running buffer consisted of 25 mM Tris, 192 mM glycine, and 1 g/L SDS. SDS–PAGE was performed using a DYCZ electrophoresis system (Liuyi Biotechnology, Beijing, China). After electrophoresis, SDS–PAGE gels were stained with Coomassie Brilliant Blue R-250 staining solution containing 1 g/L Coomassie Brilliant Blue R-250, 50% (*v*/*v*) methanol, and 10% (*v*/*v*) acetic acid for 1 h at room temperature. The gels were then destained using a destaining solution containing 40% (*v*/*v*) methanol and 10% (*v*/*v*) acetic acid until clear protein bands were visible. Gel images were captured using a scanner.

### 2.2. Preparation of SSPP^5CSK305C^-SH Mutant

To generate pGEX6P^4CS^-1, the GST^4CS^ fragment was constructed by overlap-extension PCR using pGEX6P-1 as the template. Briefly, four overlapping fragments, designated A, B, C, and D, were first amplified using KOD One PCR Master Mix with the following primer pairs: GST-F/GST-C1S-R for fragment A, GST-C1S-F/GST-C2S-R for fragment B, GST-C2S-F/GST-C3C4S-R for fragment C, and GST-C3C4S-F/GST-R for fragment D. After agarose gel electrophoresis, the four PCR fragments were purified by gel extraction. The purified fragments were then used together as templates for overlap-extension PCR, and the full-length GST^4CS^ fragment was amplified using GST-F/GST-R as the outer primers. In parallel, the pGEX6P-1 vector was digested with Msc I and EcoR I to obtain the linearized vector backbone. The full-length GST4CS fragment and the digested vector backbone were gel-purified and assembled using seamless cloning, resulting in the pGEX6P^4CS^-1 plasmid.

To generate pGEX6P-1-SSPP, the full-length coding sequence of *SSPP* (At5g02760) was amplified from *Arabidopsis thaliana* cDNA using the primer pair 6P-1-SSPP-F/R. In parallel, the pGEX6P-1 vector was digested with EcoR I and Xho I. The amplified *SSPP* fragment and the digested pGEX6P-1 vector backbone were gel-purified and assembled using seamless cloning to obtain the pGEX6P-1-SSPP plasmid. The resulting construct was confirmed by Sanger sequencing.

To generate pGEX6P-1-SSPP^5CSK305C^, the plasmid pGEX6P-1-SSPP containing the *SSPP* coding sequence was used as the template. Four overlapping *SSPP* mutant fragments, designated A, B, C, and D, were amplified using KOD One PCR Master Mix with the following primer pairs: GST-SSPPC5S-F/C54S-R for fragment A, C54S-F/C142143148S-R for fragment B, C142143148S-F/K305C-R for fragment C, and K305-F/GST-SSPP-R for fragment D. After gel purification, the four fragments were used together as templates for overlap-extension PCR, and the full-length *SSPP^5CSK305C^* fragment was amplified using GST-SSPPC5S-F/GST-SSPP-R as the outer primers. In parallel, the pGEX6P-1 vector was digested with EcoR I and Xho I. The amplified *SSPP^5CSK305C^* fragment and the digested vector backbone were gel-purified and assembled using seamless cloning to obtain the pGEX6P-1-SSPP^5CSK305C^ plasmid.

To generate pGEX6P^4CS^-1-SSPP^5CSK305C^, the pGEX6P^4CS^-1 plasmid was digested with EcoR I and Xho I, and the large vector fragment was retained. The pGEX6P-1-SSPP^5CSK305C^ plasmid was also digested with EcoR I and Xho I, and the SSPP^5CSK305C^ insert was recovered. The purified vector backbone and insert fragment were then assembled using seamless cloning to obtain the pGEX6P^4CS−1^-SSPP^5CSK305C^ plasmid.

To generate pGEX6P^4CS^-1-L-SSPP^5CSK305C^, the Linker-SSPP^5CSK305C^ fragment was amplified from the previously constructed pGEX6P^4CS^-1-SSPP^5CSK305C^ plasmid using the primer pair 4CS-L-K305C-F/4CS-L-K305C-R, with the forward primer introducing a flexible linker sequence encoding (GGGGS)_3_. In parallel, the pGEX6P^4CS^-1 vector was digested with EcoR I and Xho I. The amplified linker-containing *SSPP^5CSK305C^* fragment and the digested pGEX6P^4CS^-1 vector backbone were gel-purified and assembled using seamless cloning, resulting in the pGEX6P^4CS^-1-L-SSPP^5CSK305C^ plasmid.

In the reference construct GST^4CS^-SSPP^5CSK305C^, no additional flexible linker was introduced between GST^4CS^ and SSP^P5CSK305C^, except for the native pGEX6P-1 junction containing the HRV 3C/PreScission protease cleavage site. In GST^4CS^-L-SSPP^5CSK305C^, a flexible (GGGGS)_3_ linker was inserted immediately downstream of the HRV 3C cleavage site.

All PCR amplifications were performed using the reaction system and cycling conditions described above. Gel purification was performed using an Axygen^®^ AxyPrep DNA Gel Extraction Kit (AP-GX-50), and seamless cloning was performed using a Beyotime Seamless Cloning Kit (D7010S) according to the manufacturers’ instructions. All plasmids were confirmed by Sanger sequencing (BGI Genomics, Beijing, China). The primer sequences used for plasmid construction are listed in Supplementary Table S1.

Protein purification followed the same protocol as above, with the following modifications:

- Purification used 300 μL glutathione affinity resin (Cytiva).
- LB medium contained 100 mg/L Amp + 17 mg/L Chl.
- Lysis buffer: 1× PBS.
- Elution buffer: 50 mM Tris-HCl (pH 8.0) + 10 mM reduced glutathione.
- The protein concentration reaches at least 1 mg/mL.

### 2.3. Ligation of Ubiquitin^G76C^ and SSPP^5CSK305C^

#### 2.3.1. Dialysis Membrane Preparation

A 5 cm segment of 3.5 kDa MWCO dialysis tubing (Beyotime FDM303-5m) was boiled in NaHCO_3_ (20 g/L) + 1 mM ethylenediaminetetraacetic acid (EDTA) (pH 8.0) for 10 min, rinsed with ddH_2_O, and leak-tested.

#### 2.3.2. Dialysis Setup

Dialysis was performed in a 2 L beaker containing dialysis buffer (50 mM Tris-HCl, pH 7.5, 150 mM NaCl, 5 mM MgCl_2_, and 20 μM CuCl_2_) at 4 °C with gentle magnetic stirring. The stirring speed was adjusted to maintain the dialysis bag suspended in the buffer during incubation.

#### 2.3.3. Ligation Reaction

GST^4CS^-L-SSPP^5CSK305C^ and His-Ubiquitin^G76C^ were mixed at a 1:10 molar ratio and loaded into the dialysis bag.

#### 2.3.4. Dialysis

Dialysis was performed in a 2 L beaker containing dialysis buffer at 4 °C with gentle magnetic stirring using a PTFE-coated magnetic stir bar (DLAB Scientific Co., Ltd., Beijing, China). The stirring speed was adjusted empirically to keep the dialysis bag suspended in the buffer without contacting the bottom or side wall of the beaker. Samples were collected at 2-h intervals in the absence of β-mercaptoethanol, and the ligation efficiency was quantified through a densitometric analysis of the SDS-PAGE gels. Fresh His-Ubiquitin^G76C^ was added each time, and the buffer was replaced with 2 L of fresh solution. This was repeated three times, with overnight dialysis in the final step. During the conjugation reaction, Ub-SH was supplemented three times, with each addition corresponding to a 10-fold molar excess relative to SSPP^5CSK305C^-SH.

#### 2.3.5. Remove Excess Ubiquitin

Samples from the conjugation reactions were subjected to five rounds of ultrafiltration using a 30 kDa ultrafiltration device (Amicon^®^ Ultra 30K, Burlington, MA, USA) to reduce excess unconjugated ubiquitin, resulting in relatively purified samples for subsequent analysis.

#### 2.3.6. Analysis

Proteins were separated on 12% SDS–polyacrylamide gels. Protein staining was performed as described above. For Western blot analysis, proteins were transferred onto Amersham™ Hybond™ P 0.45 μm PVDF blotting membranes (Cytiva) using a wet transfer system (Bio-Rad, Hercules, CA, USA). TBST buffer consisted of 20 mM Tris-HCl, pH 7.5, 150 mM NaCl, and 0.1% (*v*/*v*) Tween-20. After transfer, the membranes were washed three times with TBST and then blocked with 5% non-fat milk in TBST for 1 h at room temperature. The membranes were subsequently incubated with the indicated primary antibodies, washed three times with TBST, and then incubated with HRP-conjugated secondary antibodies. After three additional washes with TBST, chemiluminescent signals were developed using Amersham™ ECL Select™ Western Blotting Detection Reagent (Cytiva, RPN2235) according to the manufacturer’s instructions and captured using a Tanon 5200 chemiluminescence/fluorescence imaging system. Band intensities were quantified using ImageJ software version 1.54g (National Institutes of Health, Bethesda, MD, USA) [26]. Figures and plots were generated using GraphPad Prism version 10 software (GraphPad Software, Boston, MA, USA).

Note: Antibiotic abbreviations: Amp (ampicillin), Kan (kanamycin), Chl (chloramphenicol). Antibody: anti-GST (1:2000; Cell Signaling Technology (Danvers, MA, USA), clone 26H1) and anti-Ubiquitin (1:10,000; Invitrogen (Carlsbad, CA, USA), clone Ubi-1, 13-1600). HRP-conjugated secondary antibodies included Goat Anti-Rabbit IgG H&L (HRP) (1:10,000; Abcam (Cambridge, UK), ab6721) and Rabbit Anti-Mouse IgG H&L (HRP) (1:10,000; Abcam, ab6728).

### 2.4. Protein Structure Prediction and Comparison

The amino acid sequences of SSPP (retrieved from the NCBI database) and its variant SSPP^5CSK305C^ were subjected to structure prediction using AlphaFold3 (https://alphafoldserver.com/; accessed on 21 April 2025). For each sequence, five models were generated. The top-ranked model, based on AlphaFold3’s internal confidence metrics (e.g., pLDDT and PAE), was selected for downstream analyses and experimental design. Global Cα root-mean-square deviation (RMSD) values were calculated using the alignment tool in PyMOL 3.1.

### 2.5. In Vitro Phosphatase Assay

In vitro phosphatase assays were performed as described with minor modifications [27]. We added 0.2 μM GST, GST-SSPP, GST-SSPP^5CSK305C^, GST^4CS^-SSPP, GST^4CS^-L-SSPP^5CSK305C^, and mUb-GST^4CS^-L-SSPP^5CSK305C^ proteins with the phosphatase assay buffer (75 mM Tris-HCl, pH 7.6, 10 mM MnCl_2_, 100 mM NaCl, 0.5 mM EDTA, and 5 mM pNPP) to obtain a final volume of 200 μL. Absorbance at 405 nm was recorded every minute for up to 30 min using a microplate spectrophotometer (BioTek (Winooski, VT, USA), Cytation5). Absorbance at 405 nm was recorded every minute for 30 min. Under these assay conditions, the increase in absorbance remained linear during the measurement period, and this recording interval was sufficient for comparing phosphatase activities among the tested protein samples. The results were repeated at least three times, and similar results were obtained.

## 3. Results

**Figure 1:**
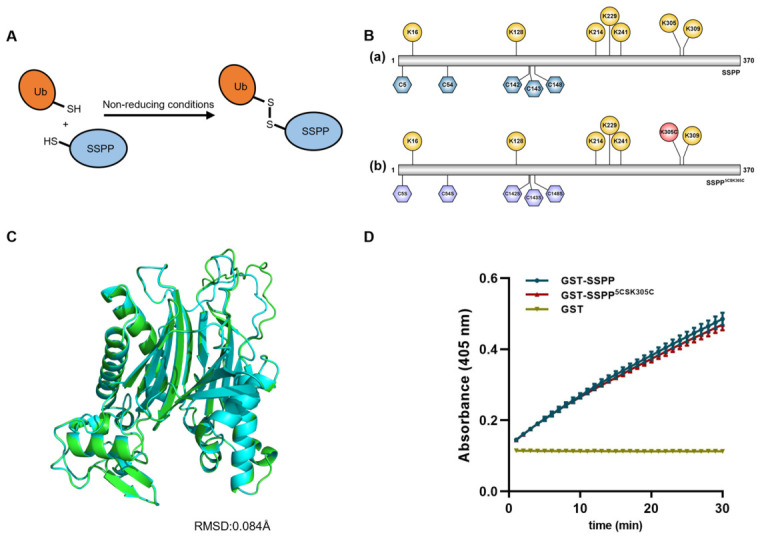
Site-specific mutagenesis of SSPP (blue) for covalent ubiquitin–substrate conjugation. (**A**) Schematic of chemical synthesis. Ub: ubiquitin (orange); SH: sulfhydryl group. (**B**) The monoubiquitination sites and cysteine residues in the wild-type SSPP (**a**) and its mutated form (**b**). Seven lysine residues (K16, K128, K214, K229, K241, K305, and K309) and five cysteine residues (C5, C54, C142, C143, and C148) are indicated by yellow circles and blue hexagons, respectively. SSPP^5CSK305C^ represents a variant of SSPP in which all five cysteine residues were mutated to serine to avoid non-specific interaction during monoubiquitylation while the K305 residue was substituted with cysteine (C, red). (**C**) Superposition of SSPP and SSPP^5CSK305C^. Protein structures were predicted by AlphaFold 3 and aligned using Cα atoms. The wild-type SSPP is colored in green, and the mutant SSPP^5CSK305C^ is shown in cyan. RMSD, root-mean-square deviation. (**D**) In vitro phosphatase assay. Phosphatase activity was assessed using 0.2 μM GST–SSPP or GST–SSPP^5CSK305C^ in the reaction buffer containing pNPP, and absorbance at 405 nm was recorded. The GST protein served as the negative control. The measurements were performed in triplicate, and the data shown represent the mean ± standard deviation (SD) of a representative experiment.

As we previously demonstrated, the low-solubility protein SSPP contains multiple monoubiquitination sites, including K16, K128, K214, K229, K241, K305, and K309 [25]. To optimize the chemical monoubiquitination system, we used SSPP as a model substrate. Among the previously reported approaches for generating ubiquitin–protein covalent linkages, we adopted the chemical conjugation method of Shin et al. [19], which involves forming a disulfide bond in place of the isopeptide bond (Figure 1A). This was achieved using the K-to-C mutant of SSPP (K305 was chosen) and the C-terminal G-to-C mutant of ubiquitin (G76C). Additionally, to prevent non-specific disulfide bond formation, five native cysteine residues in SSPP were mutated to serine (C5S, C54S, C142S, C143S, and C148S), resulting in the variant SSPP^5CSK305C^ (Figure 1B). To investigate whether the mutations affect the protein structure of SSPP, the tertiary structures of both wild-type and the mutated form of SSPP (SSPP^5CSK305C^) were predicted using AlphaFold3, an artificial intelligence system developed by DeepMind for protein structure prediction [28,29]. The two structures were aligned using Cα atoms (Figure 1C), resulting in a root-mean-square deviation (RMSD) of 0.084 Å across 308 residues, which indicates nearly identical conformations. To assess the impact of the C-to-S mutations on the phosphatase activity of SSPP, we measured the activity of the wild-type and mutant proteins using *p*-nitrophenyl phosphate (pNPP) as a substrate. To facilitate protein purification, both SSPP and SSPP^5CSK305C^ were expressed as N-terminal glutathione-S-transferase (GST) fusions, a strategy reported previously [30], because the His-tagged variant demonstrated markedly inferior purification efficiency compared to the GST fusion (Supplementary Figure S1). As shown in Figure 1D, GST-SSPP and GST-SSPP^5CSK305C^ exhibited comparable phosphatase activities toward pNPP, indicating that these mutations do not impair SSPP catalytic function.

**Figure 2:**
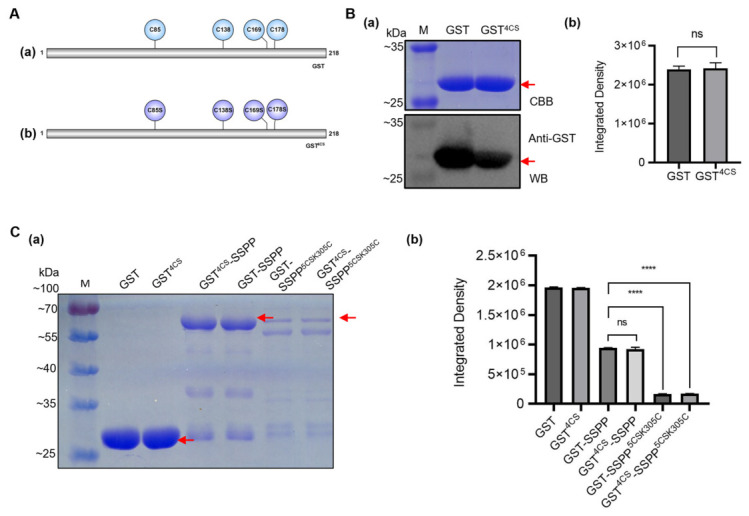
Optimizing chemical synthesis by addressing GST tag limitations. (**A**) A simplified diagram of the wild-type GST (**a**) and the engineered GST mutant (**b**). The four cysteine residues in the wild-type GST were indicated and replaced with serine residues to generate the GST^4CS^ mutant. (**B**) Comparison of protein purification yields between GST and GST^4CS^. (**a**) Proteins were purified using Glutathione Sepharose™ 4 Fast Flow and eluted with elution buffer. Purified samples were separated via 12% SDS-PAGE followed by Western blot analysis using anti-GST antibody, and the expected bands are indicated with a red arrow. (**b**) Band intensity was quantified using ImageJ. Data are shown as mean ± standard error (SE) from three biological replicates. Statistical analysis was performed using Student’s *t*-test (*p* < 0.05). ns, non-significant. M, marker. (**C**) Low solubility of GST-SSPP^5CSK305C^ and GST^4CS^-SSPP^5CSK305C^. SSPP or SSPP^5CSK305C^ was cloned into the pGEX6p-1 and pGEX6p^4CS^-1vectors. (**a**) The indicated proteins were purified using Glutathione Sepharose™ 4 Fast Flow and eluted with elution buffer. Purified samples were separated via 12% SDS-PAGE, and the expected bands are indicated with a red arrow. (**b**) Band intensity was quantified using ImageJ. Data are shown as mean ± SE from three biological replicates. Stars indicate significant differences between two samples based on Student’s *t*-test (**** *p* < 0.0001). The results revealed significantly reduced yields for GST-SSPP^5CSK305C^ and GST^4CS^-SSPP^5CSK305C^ fusion proteins compared to GST, GST^4CS^, GST-SSPP, and GST^4CS^-SSPP, indicating that the site-directed mutations in SSPP significantly impaired its protein solubility.

Although the GST tag greatly improved SSPP purification, its intrinsic cysteine residues proved problematic for subsequent chemical synthesis. To address this issue, all four native cysteine residues in GST, including C85, C138, C169, and C178, were mutated to serine, and the resultant mutant was designated as GST^4CS^ (Figure 2A). To ascertain the effect of these mutations on protein purification, Glutathione Sepharose 4 Fast Flow was utilized to purify both the wild-type GST and the mutated GST^4CS^ proteins. The results reveal that GST^4CS^ exhibited a single band corresponding to the same molecular weight as the wild-type GST and was effectively recognized using the commercially available GST antibody (Figure 2B(a)). Band intensity analysis, conducted across three biological replicates, further confirmed that the protein concentrations of GST^4CS^ and wild-type GST were indistinguishable (Figure 2B(b)). These findings indicate that GST^4CS^ retained the tag properties required for this study, including efficient purification using glutathione affinity resin and recognition by commercial anti-GST antibody.

To conduct the substrate ubiquitination assay, GST or GST^4CS^ were fused with SSPP or SSPP^5CSK305C^, respectively. As shown in Figure 2C, significantly lower concentrations of the GST-SSPP^5CSK305C^ and GST^4CS^-SSPP^5CSK305C^ fusion proteins and lower molecular weight products were observed when the purification process was carried out under the same conditions. These results suggest that the introduced mutations in SSPP adversely affect the solubility or the stability of the fusion proteins. Given the large quantity of substrate proteins required for chemical synthesis, GST^4CS^-SSPP^5CSK305C^ appeared to be unsuitable for such experimental purposes.

**Figure 3:**
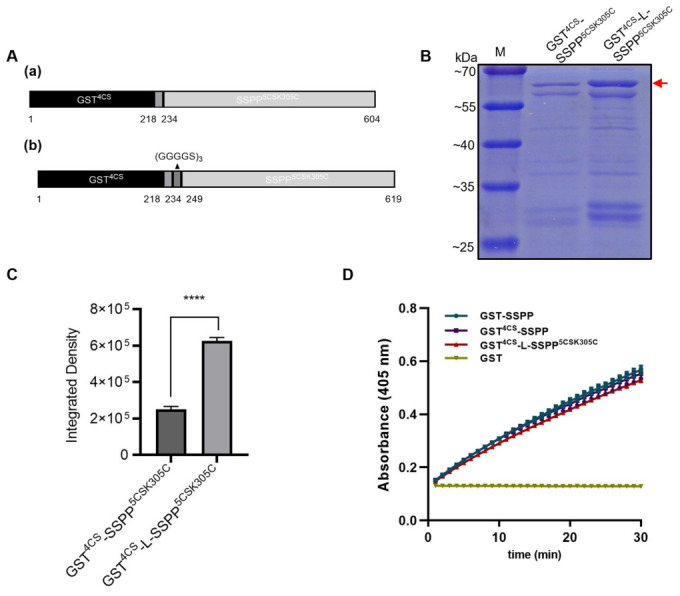
Introducing a flexible linker significantly improved the protein solubility of GST^4CS^-SSPP^5CSK305C^. (**A**) Introduction of a flexible linker between GST^4CS^ and SSPP^5CSK305C^. Schematics of (**a**) GST^4CS^-SSPP^5CSK305C^ and (**b**) GST^4CS^-GGGGS*3-SSPP^5CSK305C^ fusion proteins. The numbers below indicate amino acid positions. (**B**) Enhanced protein yield with the linker. The indicated proteins were purified using Glutathione Sepharose™ 4 Fast Flow and eluted with elution buffer. Purified samples were separated via 12% SDS-PAGE, and the expected bands are indicated with a red arrow. (**C**) Band intensity was quantified using ImageJ. Data are shown as mean ± SE from three biological replicates. Stars indicate significant differences between two samples based on Student’s *t*-test (**** *p* < 0.0001). (**D**) The phosphatase activity analysis of GST-SSPP, GST^4CS^-SSPP, and GST^4CS^-L-SSPP^5CSK305C^. The phosphatase assays using pNPP as the substrate were performed with 0.2 μM indicated proteins. The GST protein served as the negative control. The measurements were performed in triplicate, and the data shown represent the mean ± standard deviation (SD) of a representative experiment.

To address the issue of improper protein folding caused by the close spatial proximity of multiple mutations in both the GST and SSPP domains, a triple-repeat GGGGS flexible linker was strategically inserted between GST and SSPP immediately downstream of the HRV 3C (PreScission) protease site. (Figure 3A). Comparative protein purification experiments revealed that the resultant GST^4CS^-L-SSPP^5CSK305C^ variant showed a significant 2.5-fold increase in concentration relative to the GST^4CS^-SSPP^5CSK305C^ when purified under identical conditions (Figure 3B,C). This substantial improvement in protein yield underscores the effectiveness of the linker insertion in mitigating the adverse effects of the mutations and optimizing the protein for subsequent applications. We further assessed the impact of the GST^4CS^ fusion and an intervening linker on SSPP function by measuring phosphatase activity with pNPP as the substrate. The kinetic analysis revealed that GST^4CS^-SSPP and GST^4CS^-L-SSPP^5CSK305C^ had activities comparable to GST-SSPP (Figure 3D). This result confirms that the C-to-S mutations in both GST and SSPP did not impair the enzyme’s catalytic function.

**Figure 4:**
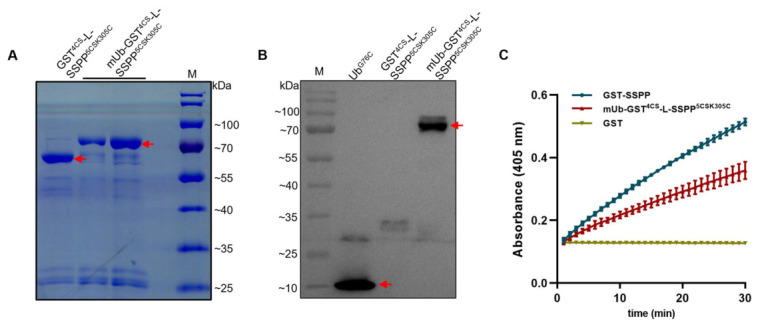
Site-specific chemical monoubiquitination of SSPP^5CSK305C^ reduces its phosphatase activity. (**A**) Chemical synthesis of mUb-GST^4CS^-L-SSPP^5CSK305C^ through the iterative addition of ubiquitin^G76C^. The GST^4CS^-L-SSPP^5CSK305C^ and final products of chemically synthesized mUb-GST^4CS^-L-SSPP^5CSK305C^ were separated via 12% (*w*/*v*) SDS-PAGE and are indicated with red arrows. The mUb-GST^4CS^-L-SSPP^5CSK305C^ products were loaded into two separate lanes, with quantities of 1.5 μg and 3 μg, respectively. M, marker. (**B**) Western blot detection of ubiquitin. Ubiquitin and mUb-GST^4CS^-L-SSPP^5CSK305C^ were detected using anti-ubiquitin antibody and are indicated with red arrows. (**C**) The phosphatase activity analysis of GST-SSPP and mUb-GST^4CS^-L-SSPP^5CSK305C^. The phosphatase assays using pNPP as the substrate were performed with 0.2 μM indicated proteins. The GST protein served as the negative control. The measurements were performed in triplicate, and the data shown represent the mean ± standard deviation (SD) of a representative experiment.

To chemically monoubiquitinate SSPP at the position of lysine 305, we crosslinked GST^4CS^-L-SSPP^5CSK305C^ with His-Ubiquitin^G76C^. Non-reducing SDS-PAGE analysis revealed a new band migrating ~10 kDa above the unmodified protein, consistent with the addition of a single ubiquitin moiety (Figure 4A). The identity of this band as monoubiquitinated SSPP was confirmed by Western blotting with an anti-ubiquitin antibody, which detected a band of identical molecular weight (Figure 4B). To determine the functional consequence of this modification, we performed the pNPP phosphatase activity assays using GST as a negative control and GST-SSPP as a positive control. As shown in Figure 4C, the monoubiquitinated protein (mUb-GST^4CS^-L-SSPP^5CSK305C^) retained robust phosphatase activity, clearly exceeding that of the GST control, but showed a modest reduction in activity compared with unmodified GST-SSPP. This result suggests that monoubiquitination at the position of lysine 305 functions as a negative regulatory modification that attenuates SSPP phosphatase activity.

## 4. Discussion

Chemical monoubiquitination is of great significance in exploring the functions of this post-translational modification. Firstly, it permits site-specific modification, thereby circumventing the heterogeneity often encountered in enzymatic methods and offering a precise research tool, as highlighted by Dhall and Chatterjee (2011) [31]. Secondly, the use of chemically synthesized monoubiquitinated proteins enables high-resolution structural studies of interaction interfaces with ubiquitin-binding domains or deubiquitinating enzymes [32]. It also offers a robust platform for exploring the impact of monoubiquitination on the formation and modification of polyubiquitin chains [33]. Finally, these synthetic proteins hold potential for mechanistic studies and biochemical characterization of ubiquitination events [34].

Traditional chemical monoubiquitination methods relying on enzymatic reactions or complex chemical coupling processes are hampered by their operational complexity, high costs, and low yields, which restrict their widespread application [16,17,18]. Additionally, the common use of GST tags for target protein purification poses further challenges. The natural cysteine residues in GST tags tend to engage in non-specific interactions with ubiquitination reagents, thereby reducing the efficiency of the synthesis. This problem is particularly pronounced for proteins with low solubility, which require intact tags to ensure adequate yields [30]. Through systematic optimization, this study successfully established a robust and cost-effective chemical monoubiquitination system that substantially addresses these technological limitations.

To address the interference caused by the four cysteine (C) residues in the GST tag, we modified the pGEX6p-1 vector by replacing these residues with serine (S), resulting in the GST^4CS^ mutant. As shown in Figure 2B, the GST^4CS^ mutant retained the same purification efficiency as the wild-type GST and was still recognized by commercial antibodies, indicating that the tag properties required for this study were retained. This substitution obviated the need for conventional tag removal steps [21,22], simplifying the workflow and notably improving the solubility of SSPP fusion protein (Figure 2C). The modified pGEX6p-1 vector can serve as a versatile tool for fusing other low-solubility target proteins in chemical monoubiquitination system, thereby circumventing the laborious process of GST tag removal. Similarly, other solubility-enhancing tags, such as maltose-binding protein [30], can undergo similar modifications to create comparable versatile vectors. These engineered vectors will not only streamline the purification process but also will enhance the overall yield and quality of the recombinant proteins, facilitating downstream applications in biochemical and structural studies.

To enable site-specific monoubiquitination of the target protein, all other cysteine residues must be mutated to serine. However, in this study, the C-to-S mutations significantly reduced the solubility of the GST-SSPP fusion protein (Figure 2C), posing a major obstacle for subsequent chemical synthesis experiment. While previous studies have shown that the production of certain proteins can be improved through sequence alterations via site-directed mutagenesis [35,36], reverting the introduced serine residues back to cysteines in this study was not feasible. Additionally, identifying other residues to mutate in order to increase solubility without affecting the structure and function of SSPP proved to be very challenging. To alleviate this issue, we strategically inserted a flexible (GGGGS)_3_ linker between the GST^4CS^ and the target protein. Following this optimization, the GST^4CS^-L-SSPP^5CSK305C^ fusion protein showed a 2.5-fold increase in purification concentration (Figure 3C), highlighting the importance of linker in enhancing the solubility of fusion proteins. This optimization provides valuable guidance for the chemical ubiquitination studies of similar low-solubility proteins, demonstrating an effective approach for overcoming purification challenges. Our results are consistent with previous reports indicating that artificial polypeptide linkers composed of glycine and serine residues provides flexibility, allows for mobility of the connecting functional domains, and improves solubility [37,38]. Flexible Gly-rich regions have also been observed as natural linkers in proteins, connecting multiple domains with loops [39]. One should be aware that the length of this artificial linker can be optimized to achieve appropriate separation of functional units or to maintain necessary inter-domain interactions. Alternatively, other flexible linkers rich in Gly and Ser but also containing additional amino acids such as Lys and Glu may also be considered to improve solubility in the future [37].

As shown in Figure 4A,B, through employing disulfide bond-mediated specific coupling, we successfully achieved precise monoubiquitination at the C305 site of the mutated SSPP protein. Interestingly, the successfully generated monoUb-SSPP exhibited reduced phosphatase activity compared with unmodified GST-SSPP (Figure 4C), indicating that monoubiquitination at the position of lysine 305 functions as a negative regulatory modification that attenuates SSPP phosphatase activity. This finding aligns with our recent work, which demonstrated that the E3 ligase ATL72 monoubiquitinates SSPP on seven possible lysine residues including K305. This modification impairs SSPP’s ability to dephosphorylate AtSARK, thereby promoting the onset of leaf senescence [25]. However, as we reported previously, the K305 residue of SSPP is not the only site ubiquitinated and the chemical monoubiquitination at other sites could be generated by precise mutation substitution [25]. Further investigation of site-specific monoubiquitination in SSPP may provide valuable insights into the identification of key monoubiquitination sites. Moreover, a complete analysis, including the sequential mutation of all lysine residues in SSPP and verification of their impact in *Arabidopsis*, is essential.

It should also be noted that, as above-mentioned, our optimized chemical synthesis system requires mutating all cysteine residues, except the specific target site, to serine to prevent non-specific conjugation of ubiquitin molecules. These necessary preparatory steps are laborious and may result in a severe reduction in the amount of protein obtainable from a given volume of bacterial culture. The strategy presented in this study is designed specifically to address such potential limitations. In addition to cysteine engineering, the introduction of flexible linkers can help to alleviate local structural constraints and improve protein folding and solubility when necessary. In particular, the linker length can be adjusted in a case-by-case manner according to the structural and biophysical characteristics of the target protein. Nevertheless, given that cysteine residues often participate in protein–protein interactions [40,41], it is necessary to first verify that the mutation from cysteine to serine does not affect the structure and function of the target protein before proceeding with the subsequent chemical synthesis experiments. In this study, we first performed AlphaFold-based structural predictions to obtain a preliminary assessment of the potential impact of C-to-S substitutions on SSPP structure, and further validated these predictions using in vitro phosphatase activity assays. As AlphaFold-based modeling provides only a computational approximation of protein structure, the combined computational and experimental evidence suggests that the C-to-S mutation does not substantially affect the structure or function of SSPP (Figure 1). Additionally, the structural analysis of the target protein can be further validated using circular dichroism experiments [42].

Another limitation of the current study is that ubiquitination efficiency was primarily estimated by SDS-PAGE densitometric analysis and Western blotting (Figure 4). Although this approach allows straightforward discrimination between ubiquitinated and unmodified SSPP species and provides a convenient assessment of relative conjugation efficiency, it does not enable precise determination of ubiquitination stoichiometry. Notably, because SSPP contains only a single engineered conjugation site (C305) and Ub^G76C^ contains only one reactive cysteine residue, the reaction scheme is designed to generate exclusively mono-ubiquitinated SSPP species. Future studies could nevertheless employ size exclusion chromatography and LC-MS/MS to further characterize product composition and validate conjugation stoichiometry with higher precision. In particular, LC-MS/MS would be required to unambiguously map the exact conjugation site and rule out alternative conjugation events. A simple way to substantiate the claim of monoubiquitination by formation of a disulfide bond would be to subject the same ubiquitinated protein sample to two SDS-PAGEs under non-reducing and reducing conditions, respectively.

In addition, we employed a conventional restriction enzyme-based cloning strategy, alternative cloning methods such as ‘Golden Gate ’ cloning [43] or seamless ligation cloning extract method [44] may provide improved efficiency and flexibility for future studies. Furthermore, while the mutated GST obtained in this work retains its solubility and utility as an immunodetectable tag, its enzymatic activity remains to be confirmed. Future work should incorporate a photometric activity assay, utilizing reduced glutathione and 1-chloro-2,4-dinitrobenzene as substrates [45], to rigorously evaluate the functional integrity of the mutated GST. Furthermore, the retention of the solubility-enhancing tags such as GST is often not compatible with a medical application of the modified protein.

Site-specific monoubiquitination of target proteins in plants remains a significant technical hurdle, particularly for low-solubility proteins recalcitrant to conventional biochemical methods. Although our in vitro protocol requires rigorous validation, it offers a crucial and reliable solution for screening and validating functional ubiquitination sites. This method provides a cost-effective and streamlined workflow by eliminating the need for cumbersome tag cleavage, thus addressing a key bottleneck in the field.

## 5. Conclusions

In summary, through the rational engineering of the GST tag, strategic insertion of a flexible linker, and thorough experimental validation, we developed an optimized platform for chemical monoubiquitination. This system effectively addresses key limitations of traditional methods, such as tag interference and protein insolubility, and offers robust technical support for investigating post-translational modifications in plant proteins. The established optimization paradigm not only enhances the feasibility of ubiquitination studies but also facilitates in vitro reconstitution of complex protein assemblies. This advancement underscores its theoretical significance and broad practical applicability, paving the way for future research in the field of protein modification and assembly.

## Data Availability

The original contributions presented in this study are included in the article and Supplementary Material. Further inquiries can be directed to the corresponding authors.

## Supplementary Materials

The following supporting information can be downloaded at: https://www.mdpi.com/article/10.3390/cimb48070666/s1.
