# Supplementary material for: Optimization of the Chemical Monoubiquitination System for Low-Solubility Protein: Achieving Balance Between Specificity and Yield
Source: Curr Issues Mol Biol. 2026 Jun 29;48(7):666. doi: 10.3390/cimb48070666 (PMC13406685; doi:10.3390/cimb48070666)
Supplement: Supplementary file 1 [file cimb-48-00666-s001.zip › Supplementary Figure S1.pdf]

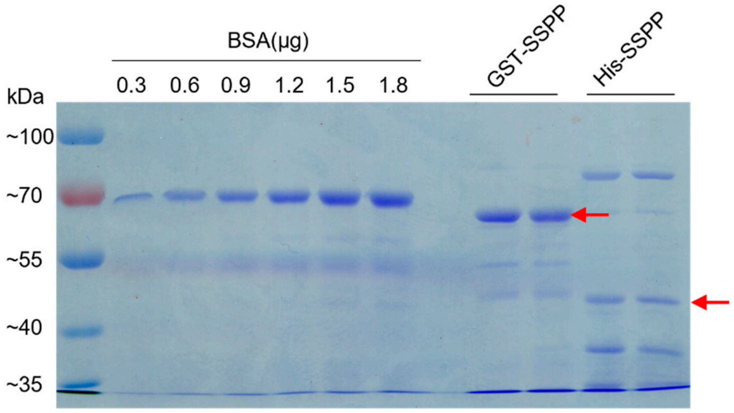

Supplementary Figure S1. Purification analysis of GST-SSPP and His-SSPP fusion proteins. Purified GST-SSPP and His-SSPP proteins were analyzed by SDS-PAGE followed by Coomassie Brilliant Blue staining. The six lanes on the left contain BSA standards, with the loading amounts indicated in the figure. A total of 1 μg protein was loaded for each GST-SSPP and His-SSPP sample lane. The red arrows indicate the target protein bands, with theoretical molecular weights of approximately 44.5 kDa for His-SSPP and 68.5 kDa for GST-SSPP.
